# Evidence for 5S rDNA Horizontal Transfer in the toadfish *Halobatrachus didactylus* (Schneider, 1801) based on the analysis of three multigene families

**DOI:** 10.1186/1471-2148-12-201

**Published:** 2012-10-07

**Authors:** Manuel A Merlo, Ismael Cross, José L Palazón, María Úbeda-Manzanaro, Carmen Sarasquete, Laureana Rebordinos

**Affiliations:** 1 Laboratorio Genética, Facultad de Ciencias del Mar y Ambientales, CACYTMAR, Universidad de Cádiz, Puerto Real (Cádiz), 11510, Spain; 2 Instituto de Ciencias Marinas de Andalucía – CSIC, Polígono Río San Pedro, Puerto Real (Cádiz), 11510, Spain; 3 Instituto de Investigaciones Cientificas, Universidad de Oriente, Isla de Margarita, Venezuela

## Abstract

**Background:**

The Batrachoididae family is a group of marine teleosts that includes several species with more complicated physiological characteristics, such as their excretory, reproductive, cardiovascular and respiratory systems. Previous studies of the 5S rDNA gene family carried out in four species from the Western Atlantic showed two types of this gene in two species but only one in the other two, under processes of concerted evolution and birth-and-death evolution with purifying selection. Here we present results of the 5S rDNA and another two gene families in *Halobatrachus didactylus*, an Eastern Atlantic species, and draw evolutionary inferences regarding the gene families. In addition we have also mapped the genes on the chromosomes by two-colour fluorescence *in situ* hybridization (FISH).

**Results:**

Two types of 5S rDNA were observed, named type α and type β. Molecular analysis of the 5S rDNA indicates that *H*. *didactylus* does not share the non-transcribed spacer (NTS) sequences with four other species of the family; therefore, it must have evolved in isolation. Amplification with the type β specific primers amplified a specific band in 9 specimens of *H*. *didactylus* and two of *Sparus aurata*. Both types showed regulatory regions and a secondary structure which mark them as functional genes. However, the U2 snRNA gene and the ITS-1 sequence showed one electrophoretic band and with one type of sequence. The U2 snRNA sequence was the most variable of the three multigene families studied. Results from two-colour FISH showed no co-localization of the gene coding from three multigene families and provided the first map of the chromosomes of the species.

**Conclusions:**

A highly significant finding was observed in the analysis of the 5S rDNA, since two such distant species as *H*. *didactylus* and *Sparus aurata* share a 5S rDNA type. This 5S rDNA type has been detected in other species belonging to the Batrachoidiformes and Perciformes orders, but not in the Pleuronectiformes and Clupeiformes orders. Two hypotheses have been outlined: one is the possible vertical permanence of the shared type in some fish lineages, and the other is the possibility of a horizontal transference event between ancient species of the Perciformes and Batrachoidiformes orders. This finding opens a new perspective in fish evolution and in the knowledge of the dynamism of the 5S rDNA. Cytogenetic analysis allowed some evolutionary trends to be roughed out, such as the progressive change in the U2 snDNA and the organization of (GATA)_n_ repeats, from dispersed to localized in one locus. The accumulation of (GATA)_n_ repeats in one chromosome pair could be implicated in the evolution of a pair of proto-sex chromosomes. This possibility could situate *H*. *didactylus* as the most highly evolved of the Batrachoididae family in terms of sex chromosome biology.

## Background

The Lusitanian toadfish (*Halobatrachus didactylus*), a member of the Batrachoididae family, is found in natural habitats of soft sand and rocky sea beds, and reefs. The Batrachoididae family comprises 71 species, belonging to three subfamilies [1] that are widely distributed across the Atlantic, Pacific, and Indian Oceans. *H*. *didactylus* is the only species of this family found in the Iberian Peninsula [2] and populations are distributed along the coasts from the Bay of Biscay (Spain) to Ghana as well as the western Mediterranean [3].

On the coasts of Portugal and southwestern Spain, this species is of moderate commercial importance. However, the real importance of this species is for research purposes rather than its commercial value. It has traditionally been used as a model animal in toxicology experiments [4], and in hematology, reproduction and histophysiology studies [5-7]. Genetic studies in the Batrachoididae family have been focused mainly on cytogenetics (reviewed by [8]); there is only one published paper in which this fish family has been studied from a molecular perspective [9]. For *H*. *didactylus* in particular, the karyotype and the localization of various repetitive sequences have been described [10].

Multigene families are comprised of multiple genes that all descend from a common ancestral gene, have similar sequences and are functionally related [11]. Traditionally, it has been presumed that the evolution of multigene families is in accordance with a model known as the “Concerted Evolution Model”, in which the members of the family are homogenized by several different mechanisms such as unequal crossing-over and gene conversion [12]. This feature makes the units show more similarity within than between related species [13]. In the last two decades, a new model has been proposed to explain controversial situations found in some multigene families: the “Birth-and-Death Evolution Model”. Under this model new genes arise by duplications during the evolution of a group of organisms, and these new genes are either fixed as functional genes or become pseudogenes [14]. The histone and the immunoglobulin gene families are typical examples of birth-and-death evolution [11].

The ribosomal genes are divided in two clusters: the major (45S rDNA) and the minor (5S rDNA) clusters. The 45S rDNA has a transcribed unit which comprises two external transcribed spacers (5’ ETS and 3’ ETS), the coding for 18S, 5.8S and 28S rRNAs, and two internal transcribed spacers, which separate the 18S rRNA from the 5.8S rRNA (ITS-1) and the 5.8S rRNA from the 28S rRNA (ITS-2); the transcribed units are separated by intergenic spacers (IGS). Meanwhile, the 5S rDNA comprises a conserved coding region of 120 bp and a non-transcribed spacer (NTS) which is variable among species in length and sequence. The two ribosomal clusters represent well-known examples of concerted evolution.

The small nuclear RNAs (snRNA) are components of the small nuclear ribonucleoprotein particles (snRNP), a complex which participate in the splicing process of the mRNA precursors [12]. Most of the snRNA are transcribed by RNA polymerase II, except for U6 snRNA which is transcribed by RNA polymerase III [15]. Within the group of fishes, there have been very few studies made of the snRNA gene family at molecular and cytogenetic level. It has been presumed that the snRNA gene families undergo a concerted evolution [11].

In this work a molecular characterization has been made of three multigene families: 5S rDNA, ITS-1 (from 45S rDNA) and the U2 snRNA gene of *H*. *didactylus*. In addition, double-FISH technique was used to ascertain the U2 snRNA gene localization and the possible co-localization of this probe with the 5S rDNA, 18S rRNA gene and (GATA)_n_ probes. The co-localization of (GATA)_n_ and the 18S rRNA gene was also tested. This technique not only provides various different cytogenetic markers, but also represents a starting point for the production of a genetic map in *H*. *didactylus*. The nucleotide variation has been estimated to assess the homogenizing forces which support the concerted evolution in the three multigene families. Finally, the results have been compared with those obtained from other members of the Batrachoididae family, and the evolutionary difference between those and *H*. *didactylus* has been established.

## Results

### Sequence analysis

After PCR amplification, two electrophoretic bands of 5S rDNA sequence could be observed, named type α and type β, and whose sizes were 420 and 200 bp respectively. The amplification with the type β specific primer gave a 190 bp band in 9 specimens of *H*. *didactylus* (data not shown). The sequencing results demonstrate that the 5S rDNA sizes varied from 413 to 424 bp in type α, and from 197 to 207 bp in type β 5S rDNA sequences. In the former the variability was due a variable poly-A region at position −71, and in the latter by a 10 bp insertion-deletion (indel) localized immediately after the poly-T termination signal. In two clones an electrophoretic band of 840 bp was obtained; this corresponded to a dimeric form of 5S rDNA type α. From these findings, it would therefore be possible to ascertain the complete sequence for 5S rDNA of type α.

**Figure 1:**
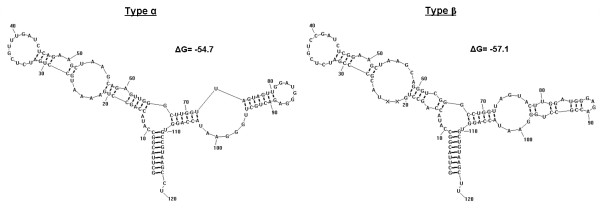
**Secondary structure prediction of the two types of 5S rRNA.** The free energy (ΔG) of the structure is shown, and was calculated considering the mean seawater temperature (15°C).

Both types of 5S rDNA sequence conserved all promoter and regulatory regions necessary for transcription by RNA polymerase III (pol III), such as the TATA-like promoter element around the position −30 upstream from the transcription starting point, the poly-T terminator region situated at the 3’ end of the coding region and the Internal Control Regions (Box A, Intermediate Element and Box C) located inside coding regions between the positions +50 to +65, +68 to +73, and +81 to +98 respectively. The RNAstructure 5.2 program showed a stable secondary structure of 5S rRNA in both cases (type α and type β) (Figure 1), with similar free energy values, and were in accordance with secondary structures predicted in previously described models [16].

**Figure 2:**
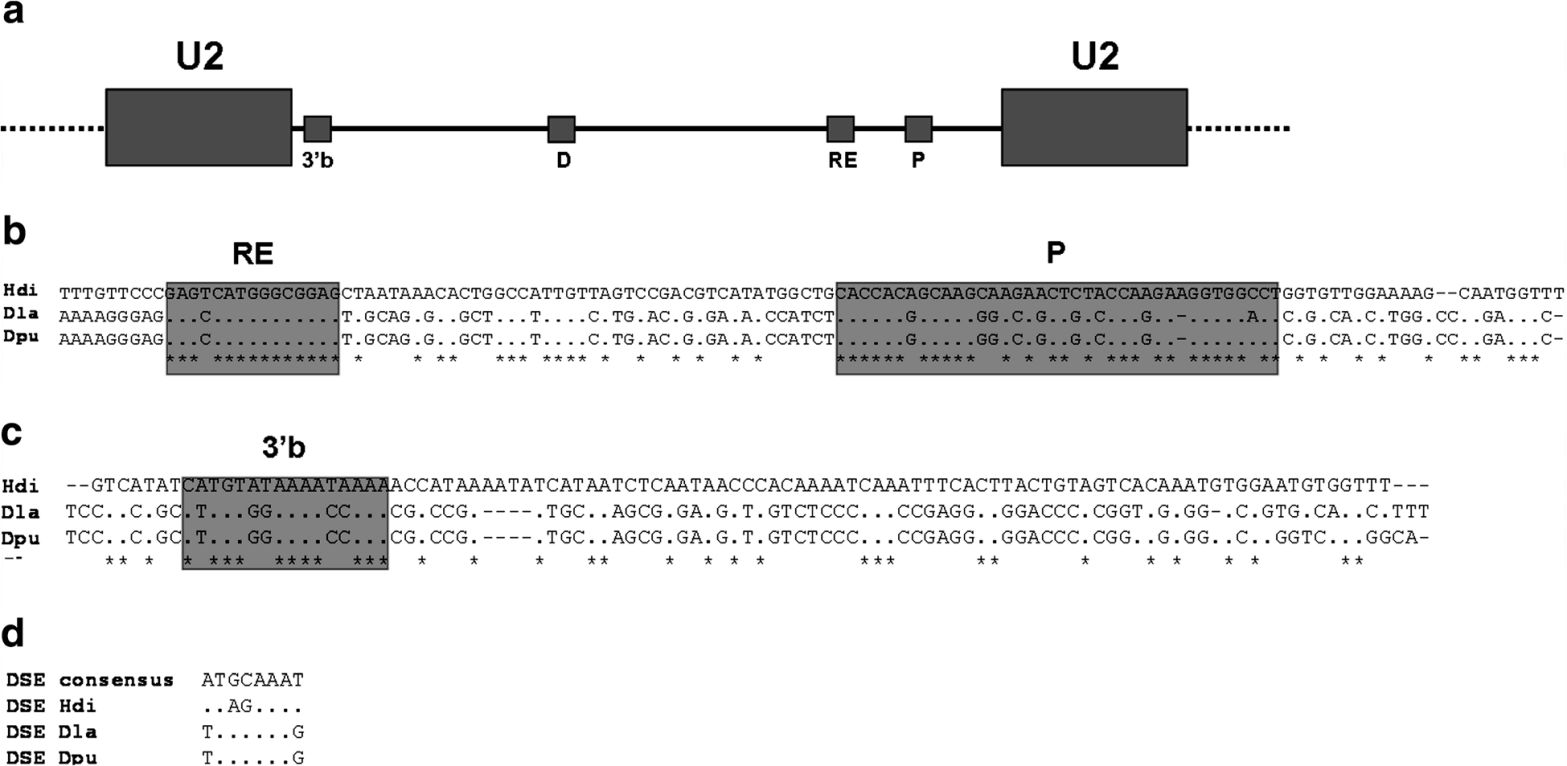
**U2 snRNA gene organization in *Halobatrachus didactylus.*** (**a**) Different regions of the U2 snRNA represented schematically. (**b**) 3’ end alignment of the U2 spacer; 3’b: 3’ Box; D: Putative DSE element; RE: Regulator Element; P: PSE element. (**c**) 5' end alignment of the U2 spacer; Hdi: *Halobatrachus didactylus*; Dla: *Dicentrarchus labrax*; Dpu: *Dicentrarchus punctatus*. (**d**) Alignment of the DSE element with those obtained from other species.

Meanwhile, the ITS-1 and U2 snDNA sequences showed one electrophoretic band of 650 and 800 bp respectively. The sequencing results demonstrated that the ITS-1 sequence presented an exact size of 664 bp, and 820 bp in the U2 snDNA sequence. Furthermore, three putative promoter and regulator sequences in the U2 snRNA gene have been found (Figure 2). These include the Proximal Sequence Element (PSE), the Distal Sequence Element (DSE) and the 3’ box within the spacer region. The PSE and 3’ box were located by alignment with consensus sequences obtained from the Moronidae species, *Dicentrarchus labrax* and *Dicentrarchus punctatus*. From the alignment of 126 bp upstream from the coding region two conserved regions were detected (Figure 2a). The more proximal of these is between position −60 and −23, while the more distal region comprises a 17 bp box, the sequence of which has not previously been described as a promoter or regulator region. The 100 bp downstream from the coding region was also aligned and a more conserved box was found at 8 nucleotides downstream from the 3’ end of the coding region (Figure 2b). A putative DSE octameric sequence was localized at −311 bp from the transcription starting point (Figure 2d), and it was located in direct orientation with respect to the consensus octameric sequence [17].

### Variability analysis

The three multigene families studied showed low values of nucleotide variability, in both coding regions and spacers. As expected, the spacer regions had higher values than coding regions, except in the U2 snRNA gene (Table 1). On the whole, the most variable multigene family was the U2 snDNA. The 5S rDNA type α also showed high nucleotide variability. The less variable genes were the 5S rDNA type β and the 18S rRNA-ITS-1-5.8S rRNA sequence. Furthermore, divergence values between the two types of 5S rDNA were different depending on the fragment considered in the study. Thus, the coding region showed a low value of divergence (*d*= 0.087 ± 0.028), whereas divergence in the NTS was higher (*d*= 0.751 ± 0.218).

**Table 1:** Polymorphism by gene region

**Region**	**Length** (**bp**)	**%GC**	**S**	**h**	**π**
*5S rRNA* (*α*)	120	52.50-55.83	12	10	0.018±0.003
*NTS* (*α*)	289-304	36.75-39.79	41	14	0.023±0.006
*5S rRNA* (*β*)	120	56.67-57.50	3	4	0.004±0.002
*NTS* (*β*)	77-87	38.96-43.02	4	5	0.009±0.003
*18S rRNA*	182	48.90-49.45	3	4	0.004±0.001
*ITS*-*1*	388	62.89-63.40	9	5	0.008±0.002
*5*.*8S rRNA*	93-94	50.54-51.06	1	2	0.002±0.002
*U2 snRNA*	189	35.45-38.10	12	7	0.024±0.003
*U2 spacer*	623-631	36.61-37.88	31	8	0.017±0.002

%GC: GC content; S: number of polymorphic sites; h: number of haplotypes; π: nucleotide variability.

**Figure 3:**
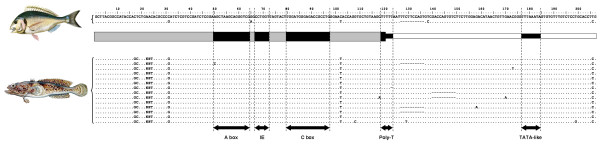
**5S rDNA type β**, ** common to *Sparus aurata *and *Halobatrachus didactylus. ***Alignment of the two clones of *Sparus aurata* (GenBank Acc. No. AY330701.1 and AY330702.1) and the type β clones of *Halobatrachus didactylus*. A schematic representation of 5S rDNA is included, highlighting the A box, the Intermediate Element (IE), the C box, the poly-T terminator region, and the TATA-like box.

**Figure 4:**
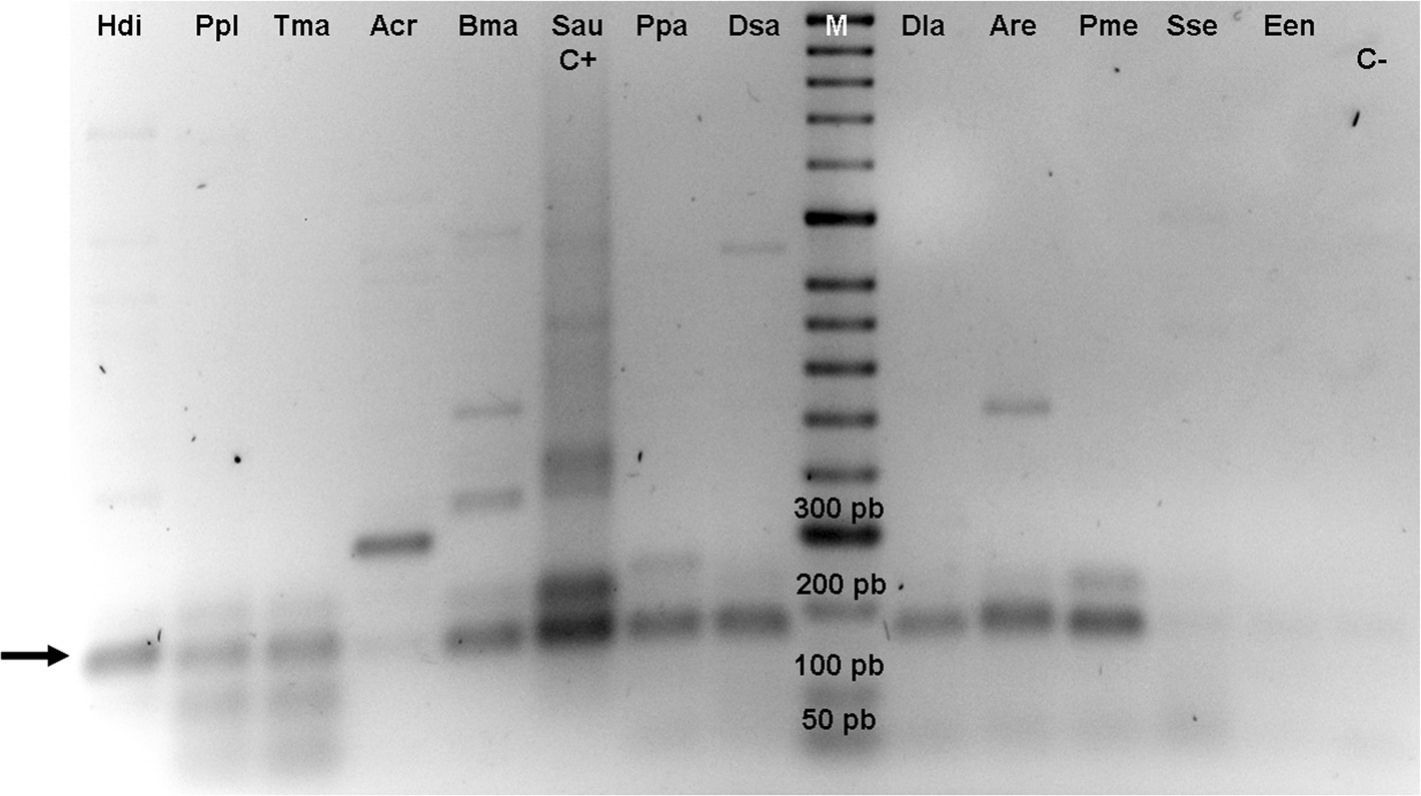
**Agarose gel electrophoresis of 5S rDNA type β species**-**specific PCR.** Positive products of 190 bp are marked by an arrow. Hdi: *Halobatrachus didactylus*; Ppl: *Porichthys plectrodon*; Tma: *Thalassophryne maculosa*; Acr: *Amphichthys cryptocentrus*; Bma: *Batrachoides manglae*; Sau: *Sparus aurata* (positive control); Ppa: *Pagrus pagrus*; Dsa: *Diplodus sargus*; Dla: *Dicentrarchus labrax*; Are: *Argyrosomus regius*; Pme: *Plectorhinchus mediterraneus*; Sse: *Solea senegalensis*; Een: *Engraulis encrasicholus*; C-: negative control.

The gene families under study were subjected to BLASTN search at the NCBI database; all coding regions (5S rRNA, 18S rRNA, 5.8S rRNA and U2 snRNA) showed high degrees of identity with others obtained from a wide variety of fish species. Spacer regions did not present any significant homologies, except for the 5S rRNA gene spacer (NTS) type β which, surprisingly, presented a high degree of homology with the NTS from *Sparus aurata* described by [18] (Figure 3). The analysis with the type β demonstrated that this type was present in all specimens of *H*. *didactylus* studied, as well as in all the species included for this experiment (see Methods section), except in *Solea senegalensis* and *Engraulis encrasicholus* (Figure 4). However, these two species showed positive bands in the parallel experiment with ITS-1 primers.

### Phylogenetic analysis

**Figure 5:**
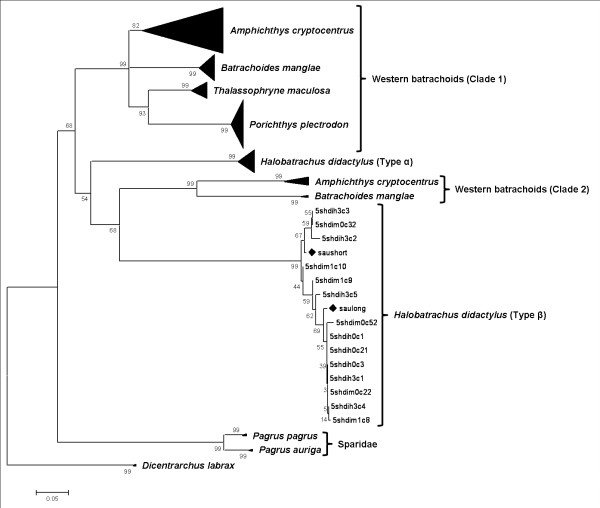
**Phylogenetic Maximum Likelihood tree of NTS sequences based on GTR substitution model.** Type β clones of *Halobatrachus didactylus* are shown. Western batrachoid clones have been collapsed. *Dicentrarchus labrax* clones have been rooted as an outgroup. *Sparus aurata* clones are also shown, preceded by a rhombus.

The Maximum Likelihood (ML) tree obtained (Figure 5) was compared with the Neighbor-Joining (NJ) tree previously described by [9] for other species of the Batrachoididae family (Western batrachoids). As in the NJ tree, the ML tree divided the Western batrachoids species in two clades, characterized by different NTS types. In the clade 2 (following the nomenclature devised by [9]) of these species, the types α and β of *H*. *didactylus* were grouped on the same branch, but within that branch, the type β clade formed a monophyletic group with the clade 2 of the Western batrachoids. The two sequences of *S*. *aurata* mentioned earlier were clustered within the *H*. *didactylus* type β clade with a high branch support. Therefore, *S*. *aurata* did not group with the two other sparid species, which formed a separate cluster from that of the Batrachoididae. Within clade 1 of the Western batrachoids, the clones of the species *Amphichthys cryptocentrus* were clustered in the same group, whereas in the NJ tree previously described by [9], these clones were divided into two groups.

### Cytogenetic analysis

**Figure 6:**
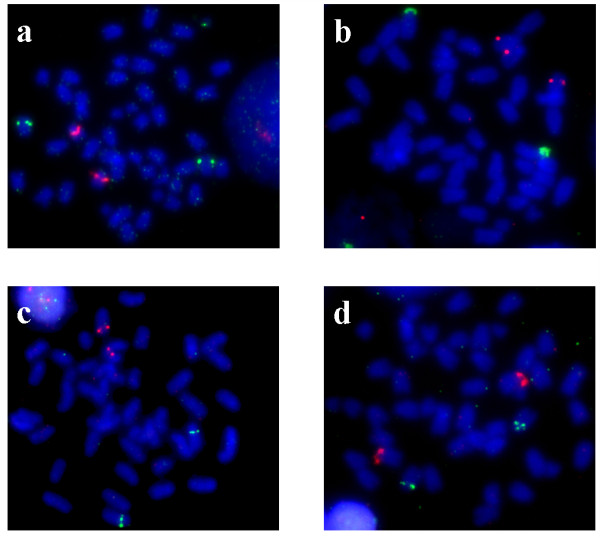
**Metaphase of *Halobatrachus didactylus* after FISH treatment.** The following probes were used: (**a**) U2 snRNA gene (green signals) versus 5S rDNA (red signals); (**b**) 18S rRNA gene (green signals) versus U2 snRNA gene (red signals); (**c**) (GATA)n sequence (green signals) versus U2 snRNA gene (red signals); (**d**) (GATA)n sequence (green signals) versus 18S rRNA (red signals).

Four double-FISH experiments were carried out with the following: the U2 snRNA gene with 5S rDNA (Figure 6a); the 18S rRNA gene with U2 snRNA gene (Figure 6b); the (GATA)_n_ sequence with U2 snRNA gene (Figure 6c); and the (GATA)_n_ sequence with 18S rRNA gene (Figure 6d). The 5S rDNA probe only includes the type α. The double-FISH treatments with 5S-18S and 5S-GATA probes had been performed in previous work [10]. The U2 snDNA probe was localized in the middle of the long arm of a medium-size submeta/subtelocentric chromosome pair. No co-localization between the U2 snRNA gene probe and the remaining probes was detected. Double-FISH treatment with (GATA)_n_ and 18S rRNA gene probes showed a non co-localized pattern.

## Discussion

Two types of 5S rDNA have been found in *H*. *didactylus*, named here as type α and type β. The NTS sequences between the two types do not share significant homologies. Furthermore, the degree of divergence of the coding region between the two types is more than 5 times higher than the nucleotide variability within the coding region of type α, and more than 28 times higher than such variability in type β (Table 1). Both types presented all the regulatory elements which make them functional genes. The existence of two types of 5S rDNA appears to be common in fish species [9,19,20], and this situation has been commonly explained as a dual expression system, where one type is expressed in both somatic and oocyte cells and the other type only in oocyte cells [21].

In a previous study [9] with four species of the Batrachoididae family from the Western Atlantic, it has been found that two species also shared two types of 5S rDNA, and the two other species have only one type. Therefore, it cannot be concluded that a dual system of 5S rDNA is generally established in the Batrachoididae family. In the four Western Atlantic species the NTS sequences are each similar to the others (see Figure 3 of [9]), whereas in *H*. *didactylus* these sequences are distantly related to those of the four species. This feature could indicate that *H*. *didactylus* has evolved independently from the Western Atlantic species, due to the geographic isolation between them, which could have contributed to the greater divergence found between Eastern and Western Atlantic species.

The sequences of both coding regions and the NTSs are quite conserved in *H*. *didactylus*, and the secondary structure agrees with previous described models [16] (Figure 1). Therefore, concerted evolution is the more feasible model which is acting in each locus of this multigene family. Under this model, the different units of the gene family tend to homogenize their sequences by means of mechanisms such as unequal cross-over and gene conversion [12], thus spreading mutation events along the multigene family. Concerted evolution makes the sequence more similar within the species than it is among related species [13]. Traditionally it has been established that the 5S rDNA gene family is a paradigm for the concerted evolution model; however, although this is the most common model observed for this gene, in more and more studies that are published, it is concluded that the 5S rDNA gene family can follow a different evolution model. Some authors have described 5S rDNA gene families which fit better with a birth-and-death evolution model than with the concerted model [9,14,22], or with a mixed model with elements of both birth-and-death and concerted evolution [23]. In the *H*. *didactylus* case, although the concerted model is acting at locus level, at genome level, the presence of two types and the between-species clustering of the multigene family indicate a birth-and-death evolution model.

As mentioned above, surprisingly the 5S rDNA type β presented homology in the NTS sequence with those obtained in *S*. *aurata*[18] (Figure 3). This species and *H*. *didactylus* belong to different superorders (Acanthopterygii and Paracanthopterygii respectively), which diverged 55 My ago [24]. Both species could have inherited the type β from an ancestral species common to the two supeorders, and could have maintained in some lineages and lost in others along the fish evolution. Using the type β specific primer, we were able to conclude that this sequence is found in all specimens studied of *H*. *didactylus*, as well as in *S*. *aurata* and the remaining species belonging to Batrachoidiformes (*Porichthys plectrodon*, *Thalassophryne maculosa*, *Amphichthys cryptocentrus* and *Batrachoides manglae*) and Perciformes order (*Pagrus pagrus*, *Diplodus sargus*, *Dicentrarchus labrax*, *Argyrosomus regius* and *Plectorhinchus mediterraneus*) (Figure 4). However, the β type is not present in either the Pleuronectiformes (*S*. *senegalensis*) or Clupeiformes orders (*E*. *encrasicholus*). Nevertheless, taking into account the highly dynamism to which the NTS are subjected, and the period elapsed since the two superorders began to diverge, another theory could be applied: a Horizontal Transference (HT) between ancient species of Perciformes and Batrachoidiformes groups. Therefore, the new type found in *H*. *didactylus* was not generated by a duplication within the own genome, but rather by an “extragenomic duplication”.

The HT phenomenon has been documented extensively in prokaryotes, and is a well-known mechanism of gene exchange [25]; it has been assumed that between 88% and 98% of the expansion of protein families in prokaryotes is due to HT [26]. However, the occurrence of HT between eukaryote organisms remains obscure [27]. It has been postulated that HT is a very important mechanism, the source for much biological innovation; indeed, some of the most significant events in cell evolution have been associated with HT, such as the origin of the primordial eukaryote cell, and HT has contributed to the characteristics of the multicellularity [28]. All documented cases of HT between eukaryotes involve transposable elements (TE), since their ability to mobilize and integrate within a genome makes them susceptible to horizontal transposable transference (HTT) [29]. Cases have been reported of HTT between a wide range of eukaryotes [30-34]. To date, the transfer of host genes by TEs between different eukaryote species has not yet been observed, but it is known that TEs are able to capture and transduce sequences with considerable frequency within a species [35]. Thus, HTT could be the mechanism responsible for a lateral movement of genes between eukaryotes.

It has been demonstrated that 5S rRNA genes and retro-transposons can interact with one another [36], and this interaction might be the cause of the pattern of evolution and the dispersed arrangement of some fungal organisms [37]. Similarly, this interaction could “open the door” for a 5S rRNA gene lateral transfer. The precise mechanism whereby a sequence is transferred horizontally from one species to another remains elusive, although various different mechanisms have been proposed in which some particles or organisms could take part as an intermediate, such as a virus [38], a bacteria [39] or a parasitic organism [40]. However, it is difficult to demonstrate the involvement of such a microorganism and virus, because of their capacity to erase any trace of non-essential DNA [28]. Few cases of HT from eukaryotes to virus [41] or bacteria [42] have been described. Typically, there are different ways to detect HT: (i) the high degree of similarity in sequences between two species phylogenetically distant; (ii) the rate of nucleotide variability of a transferred gene might be lower than that of the other orthologous genes; and (iii) in a phylogenetic analysis there should be incongruence between the phylogenies of the gene and of the organism [27]. The first way is the one used most often to ascertain the HT [28], and similarity can clearly be seen in the *H*. *didactylusS*. *aurata* case. Secondly, the nucleotide diversity of the putative transferred genes (type β) is lower than that of the orthologous genes (type α). Lastly, the phylogenetic analysis supports with high branch confidence an event of HT, since the *S*. *aurata* clones and type β are strongly grouped (Figure 5), thus leading to incongruence between the taxonomic situation and the NTS-based phylogeny.

Another alternative way of horizontal transfer should not be discounted: the sperm-mediated gene transfer (SMGT). It is well-known that sperm cells are able to capture exogenous DNA and to transfer it to the oocyte at fertilization [43]. This capacity has led to sperm cells being used as vectors for transferring DNA in transgenic experiments [44-46]. The frequency of sperm-exogene DNA uptake depends on several factors, such as the presence of inhibitory glycoproteins and the maintenance of optimum conditions for the DNA uptake in terms of quantity, length and primary structure of the exogene DNA [47]. It has been established that the greater the quantity of exogenous DNA, the higher the possibility of take-up by sperm cells [47,48] reported that the DNA content in sediments is 3 to 4 orders of magnitude higher than in the water column. Taking this into account and the benthic behaviour of the majority of species of the Batrachoidiformes order, the possibility of a SMGT is plausible.

It is commonly considered that the U2 snRNA gene also evolves in a concerted fashion [11]. However, there are few studies with this gene to conclude unequivocally that the concerted evolution is the general model followed by the U2 snRNA gene. The organization of U2 snRNA has only been investigated in humans [49], primates [50] and moronid fishes [51], and the same characteristics are shared, i.e., U2 snRNA are clustered in tandem arrays and undergo a concerted evolution model. The U2 sequence homology observed in many different specimens and units has led us to confirm a similar situation with the U2 snRNA of *H*. *didactylus*. In all the studies mentioned above (including the present work) the U2 snRNA gene is localized in one chromosome pair, although the position inside the chromosome is not conserved. A dispersed gene arrangement facilitates a birth-and-death evolution model [37]; therefore a clustered arrangement could be propitious for a concerted evolution model. Moreover, taking into account the results obtained by [8] with other species from the Batrachoididae family, it is possible that there has been a transition from a birth-and-death to a concerted evolution model within this fish family, since three distinct patterns of hybridization have been seen in only five species analyzed: dispersed, clustered, and both patterns together.

Despite the scarcity of studies describing the molecular characterization of the U2 snRNA in fish species, the only four species characterized to date, *Solea senegalensis*[12], *Dicentrarchus labrax* and *D*. *punctatus*[51], and *A*. *regius*[52] all showed a close linkage with another member of the U snRNA family. This linkage has been applied as a useful tool for the identification of different species of sole [53]. However, in *H*. *didactylus*, the U2 was not found in linkage with any other U snRNA; this absence is, so far, the only instance found within a fish group. There are insufficient data to conclude whether the U2 linkage in fishes is a general rule or is an exceptional case of only some fish families. In either case, the analysis of this gene family could be an interesting tool for evolutionary and phylogenetic purposes.

The search for promoter elements in the U2 spacer regions yielded four putative regions (Figure 2). The nearest to the 5’ coding region was located at −60 bp, and, as described by other authors [15,54], the PSE element is located between positions −60 and −50; therefore, the box found at position −60 could be the PSE regulatory element. A putative 3’ box was also localized at 8 nt downstream from the coding region, which is close to the localization described by [54] for this box (9 nt downstream). The DSE enhancer element is independent of the position and orientation, so these characteristics make it difficult to find a possible DSE by aligning with other sequences. Despite this, it has been reported that the DSE element is usually found around 200–250 bp upstream from the PSE [55]. A putative DSE element has been observed in *H*. *didactylus* at −244 bp upstream from the PSE, so that could be the enhancer element. The presence of all these regulator elements favours the supposition of a transcriptionally active gene.

The ITS-1 region without 18S and 5.8S coding regions comprises 388 bp, and the GC content is between 62.89% and 63.40%. Although this GC content is similar to the average for the Osteichthyes group (68.0% ± 4.2), the ITS-1 size is one of the smallest within this group, which varies between 318 and 1518 bp [56]. When the ITS-1 sequence was subjected to BLASTN search, an ITS-1 fragment aligned with another from many fish species, reflecting the moderate nucleotide conservation of this sequence compared with other spacer regions (IGS, NTS, etc.). This region undergoes some selective pressure, because their secondary structures are necessary for the rRNA maturation process [57]. These features make this sequence suitable for phylogenetic analysis at the species level.

The relatively low value of the nucleotide variability in the spacer regions of the three sequences treated here (Table 1) leads us to accept that their evolution has been according to the concerted model. Unequal cross-over and gene conversion are the common mechanisms that the multigene families undergo with a concerted evolution model. However, coding regions undergo another additional mechanism, such as the purifying selection, which makes them less variable. The differences in the variability found in the coding region in each gene could be due to opposing differences between homogenizing and diversifying forces. Therefore, the dynamic of the concerted evolution process depends on two conflicting forces: the homogenizing forces and the forces which generate variability [58]. It has been postulated that the unequal cross-over and gene conversion mechanisms occur more frequently in chromosome regions adjacent to the telomeres [11], thus increasing the effectiveness of concerted evolution. On this point, cytogenetics data support the conclusions already obtained with nucleotide diversity, i.e., the concerted mechanisms are more frequent in the multigene family which is situated in the telomeric position, as is the 18S rRNA, resulting in less nucleotide variation. Conversely, the two most variable multigene families (5S rDNA and U2 snRNA gene) are localized in internal positions.

None of the four probes used to hybridize in *H*. *didactylus* chromosomes were co-localized (Figure 6; Table 2); therefore they represent four different chromosomal markers, which is useful in a preliminary genome mapping. Comparisons with similar results obtained from other species of Batrachoididae family offer some evolutionary trends (Table 2; see also [8] and [10]). The five implicated species show a variable fundamental number (FN), which implies that large chromosomal re-arrangement events have occurred within this fish family, the most recent species being that with the highest FN [59]. Taking this into account, the most recent species is *H*. *didactylus* (FN= 72; [10]). Comparing the FISH results obtained in the present study with those from the other Batrachoididae species [8], some further relevant comments can be made. Firstly, U2 snRNA and (GATA)_n_ sequences have been gradually changing from a dispersed organization throughout the genome, to become localized in a single locus as in *H*. *didactylus*. Second, the ancestral (plesiomorphic) localization for 5S rDNA in the Batrachoididae family is in only one chromosome pair and in an internal position of the long arm (q arm), because this organization has been observed in 3 out of the 5 species. Finally, the major ribosomal probe shows the most conservative pattern, which also matches with the plesiomorphic condition in the fish group, i.e., in an internal position and near the centromere [10].

**Table 2:** **Localization summary of the five probes used in*Halobatrachus didactylus*and other Batrachoididae species**

**Species**	**2n**	**FN**	(**GATA**)_**n**_	**5S rDNA**	**18S rRNA**	**U2 snRNA**	**Gene co**-**localization**
*Halobatrachus didactylus* (present work)	46	72	1 SMT (qSc)	1 SMT (qSc)	1 SMT (pTl)	1 SMT (qI)	None
*Batrachoides manglae*[8]	46	48	Dispersed (++)	2 A (1 qStl and 1 qSc)	1 STL (pTl)	Dispersed (+++) 1 A (qStl)	None
*Porichthys plectrodon*[8]	44	62	Dispersed (+++)	1 MT (qI)	1 STL (pTl)	Dispersed (+)	None
*Thalassophryne maculosa*[8]	46	64	Dispersed (+++)	1 STL (qTl)	1 STL (pTl)	Dispersed (+) 1 STL (qI)	18S rRNA and U2 snRNA
*Amphichthys cryptocentrus*[8]	46	52	Dispersed (++)	1 STL (qSc)	1 STL (pTl)	Dispersed (+)	18S rRNA and 5S rDNA

+: low abundance; ++: medium abundance; +++: high abundance; A: acrocentric; STL: subtelocentric; SMT: submetacentric; MT: metacentric; q: long arm; p: short arm; Sc: subcentromeric; I: internal; Stl; subtelomeric; Tl: telomeric.

Repetitive DNAs, like GATA repeats, are sequences with an important role in the structural and functional evolution of genomes [60]. In particular, the (GATA)_n_ sequence has been implicated in the sex chromosome development of several organisms, including humans [61], snakes [62], guppy fish [63] and wolf fish [64]. It is probable that the sex-determining genes arose from a mutation of a duplicated gene, resulting in a novel sex-determining gene and chromosome [65]. Thus, it cannot be discounted that the GATA clustering in a chromosome pair observed in *H*. *didactylus*, could be associated with a proto-sex chromosome pair.

## Conclusions

For 5S rDNA, a mixed evolutionary model has been proposed, in which the presence of two different types that group in a between-species pattern reveals a birth-and-death model, but the high homogeneity of the sequences in each type indicates a homogeneization derived by the concerted model. Moreover, the existence of the birth-and-death process could not be originated by a duplication of a pre-existing 5S rDNA; instead, an ancient HT event between Batrachoidiformes and Perciformes could have introduced the new type. The 5S rDNA provides the requirements for a HT because of its recognized ability to transpose to other loci [66]. Therefore future research on this type of transfer should also be directed towards ascertaining the specific mechanism by which the transfer took place.

The U2 snRNA gene was not co-located with the other repetitive sequence, so four different chromosomal markers have been obtained in *H*. *didactylus*, which is important for producing a preliminary physical map of the species. Moreover, cytogenetic data have shown that the U2 snRNA gene have experienced a transition from a dispersed organization to a clustered organization during the Batrachoididae evolution. A similar case has been detected for (GATA)_n_ repeats. All these characteristics make the Batrachoididae family an interesting group for studying the multigene families from the evolutionary perspective.

## Methods

### Sampling, PCR amplification, cloning and sequencing

Up to six specimens (3 males and 3 females) of *H*. *didactylus* were collected from natural populations in the Bay of Cadiz (SW Spain) for molecular purposes. Meanwhile, cytogenetic analysis was made from three specimens. Sampling procedures comply with the ARRIVE guidelines and with the UCACG052009 Regulation of the Bioethical Committee from the University of Cádiz.

Genomic DNA was isolated from muscle tissue of the six specimens using FastDNA kit® (Q-Biogene). Extraction quality was validated by electrophoresis in agarose gel (1.5%) containing 0.5 μg ml^-1^ ethidium bromide. Three individuals were used to amplify the ITS-1 and U2 snRNA multigene families, while four were amplified for 5S rDNA (Table 3). These PCR amplifications were made using the following primers: A and B described by [67] for 5S rDNA amplification; Sp18 and Sp58 described by [68] for 18S rRNA-ITS-1-5.8S rRNA amplification; and U2ang-Fwd and U2ang-Rev described by [69] for U2 snDNA amplification. An internal reverse primer specific to the NTS of the 5S rDNA type β (5SBETAINT: 5’-CGAGGTGCAGGAGACAAACAC-3’) was designed to corroborate the amplification in the following fish species: *H*. *didactylus*, *S*. *aurata* (positive controls), four species of the Batrachoididae family (*Porichthys plectrodon*, *Thalassophryne maculosa*, *Amphichthys cryptocentrus* and *Batrachoides manglae*), two species of the Sparidae family (*Pagrus pagrus* and *Diplodus sargus*), *Dicentrarchus labrax* (Moronidae family), *Argyrosomus regius* (Sciaenidae family), *Plectorhinchus mediterraneus* (Haemulidae family), *Solea senegalensis* (Soleidae family) and *Engraulis encrasicholus* (Engraulidae family). Parallel to this experiment another PCR was carried out with the ITS-1 primers in the same species, as positive control for genomic DNA. Reactions were carried out in a final volume of 50 μl containing 60–80 ng of genomic DNA, 3 mM Cl_2_Mg, 300 μM dNTP, 0.2 pmol of the forward and reverse primers and 3 U of Taq polymerase (Euroclone). The PCR conditions were according to [70] and were performed in a Gene Amp® PCR System 2700 (Applied Biosystems) thermal cycler.

**Table 3:** **Specimens used**, **number of clones sequenced** (**N**), **and GenBank accession number**

**Sequence**	**Specimen**	**N**	**GenBank acc**. **no**.
5S rDNA	♂0 (type α)	5	JN406335- JN406337; JN406339- JN406343
	♂0 (type β)	3	
	♀0 (type α)	5	JN406320- JN406326; JN406338
	♀0 (type β)	3	
	♂1 (type α)	6	JN406344- JN406352
	♂1 (type β)	3	
	♀3 (type α)	3	JN406327- JN406334
	♀3 (type β)	5	
ITS-1	♂0	3	JN406365- JN406366; JN406370
	♂2	3	JN406367- JN406369
	♀4	3	JN406362- JN406364
U2 snRNA	♂0	3	JN406356- JN406358
	♀0	3	JN406353- JN406355
	♂1	3	JN406359- JN406361

The specimens are denoted by a female or male symbol followed by a number.

The PCR products were purified using the NucleoSpin® Extract II kit (Macherey-Nagel), and cloned into pGEM®-T Easy Vector (Promega). Plasmid DNA was extracted using NucleoSpin® Plasmid (Macherey-Nagel). DNA sequencing was performed with fluorescence-labeled terminator (BigDye Terminator 3.1 Cycle Sequencing Kit; Applied Biosystems) in an ABI3100 Genetic Analyzer.

### Sequence analysis

From 7 to 9 clones per specimen were sequenced in 5S rDNA, and 3 clones per specimen in both ITS-1 and U2 snRNA gene (Table 3). Sequence data have been deposited with the GenBank Data Library under Accession Numbers JN406320 to JN406370.

The sequences were aligned in MAFFT [71] using the slow and iterative refinement method strategy (FFT-NS-i). Consensus sequences were obtained with the Bioedit software [72]. A sequence-similarity search of coding and spacers sequences from the different multigene families was performed in BLASTN [73] to determine the similarities of the sequences obtained with other sequences from the GenBank database. Promoter and regulator regions of the 5S gene were detected by comparison with models previously described [20]. Regarding the U2 snRNA gene, the PSE and 3’ box elements were detected by aligning, respectively, the 5’ end and the 3’ end (respect to the coding region) with those obtained in the Moronidae fish family [51]. The putative DSE region was detected also comparing with those of the Moronidae family and with the consensus octameric sequence described for this region (ATGCAAAT) [17]. DnaSP version 5 program [74] was used to obtain the nucleotide variability (π), the number of polymorphic sites (s) and the number of haplotypes (h) in both coding and spacer regions of the three gene families. On the other hand, evolutionary distance between 5S rDNA types was calculated, using MEGA5 program [75], by the number of base substitutions per site from averaging over all sequence pairs, using the Kimura-2-parameter [76] in relation to evolutionary distances (*d*). Standard error estimates were obtained by a bootstrap procedure (1000 replicates), and complete deletion option was also applied.

The dimers obtained from 5S rDNA type α were used to extract the complete sequence of the coding region, and this sequence was subjected to the RNAstructure 5.2 program [77]. A consensus sequence from 5S rDNA type β was also obtained to be folded in RNAstructure 5.2 program. The secondary structures obtained were compared with those predicted in previous described models [16].

### Phylogenetic analysis

A phylogenetic analysis was carried out with the NTS sequences of *H*. *didactylus* and those of other four species from the Batrachoididae family, which were extracted from GenBank database (from GU645582 to GU645701). Six sequences of two different species of the Sparidae family were also included in the analysis, two from *Pagrus pagrus* (Acc. No. HM004380 and HM004384), two from *Pagrus auriga* (Acc. No. HM004389 and HM004390) and two from *Sparus aurata* (Acc. No. AY330701 and AY330702). In addition, two sequences of *Dicentrarchus labrax* were rooted as outgroup (Acc. No. HM014364 and HM014367). A Neighbor-Joining (NJ) [77] tree was firstly inferred in MEGA5 [75]. The Branch support was tested by the bootstrap method [78]. The evolutionary distances were computed using the p-distance method [79]. This NJ tree was used thereafter as the starting tree for the inference of a Maximum-Likelihood tree in the PhyML 3.0 program [80]. Previously, a statistical selection of best-fit models of nucleotide substitution had been performed by using the jModelTest program, version 0.1.1 [81], using the Akaike Information Criterion (AIC) for model selection. The GTR model [82] was chosen as the best for the PhyML 3.0 program (−lnL= 12156.5732, AIC= 25252.6211). The branch support of the ML tree was measured by applying the non-parametric version of the approximate likelihood ratio test (SH-aLRT) [83], and the option SPR was also chosen for tree improvement.

### Cytogenetic techniques

Chromosome preparations were made from cephalic kidney as described by [2]. Slides were pretreated with RNase, pepsin and formaldehyde according to [84]. Finally, the samples were dehydrated in successive steps using ethanol and stored at −80°C up to the moment of hybridization.

5S rDNA and U2 snRNA probes were labeled by the PCR-based method using PCR primers as previously mentioned. Similarly, 18S rDNA probe was labeled by PCR-based method using the primers 18S F and 28S R described by [85]. The (GATA)_n_ probe was obtained by PCR as was described by [86], using the primers (GATA)_7_ and (TATC)_7_. The labelling of GATA probe was performed by Nick Translation procedure according to the manufacturer’s instructions.

Double-FISH techniques were performed according to [10]. Finally, better images were obtained with an epifluorescence microscope (Axioskop 2 Plus, Zeiss), equipped with a cooled camera (CoolSnap, Photometrics^Â©^ Inc.).

## Competing interests

The authors declare that they have no competing interests.

## Authors’ contributions

MAM carried out the molecular genetic studies, the sequence alignment, the phylogenetic analysis, the cytogenetic techniques, and drafted the manuscript. IC participated in the sequence and phylogenetic analysis and helped to draft the manuscript. JLP, MUM and CS carried out the sampling, tissue extraction, and chromosome preparations. LR conceived and coordinated the study, participated in its design, discussed the results and corrected the manuscript. All authors read and approved the final manuscript.
